# The impact of ABO and RhD blood types on *Babesia microti* infection

**DOI:** 10.1371/journal.pntd.0011060

**Published:** 2023-01-25

**Authors:** Ryan Philip Jajosky, Jane O’Bryan, Anne Spichler-Moffarah, Philip G. Jajosky, Peter J. Krause, Laura Tonnetti

**Affiliations:** 1 Brigham and Women’s Hospital, Boston, Massachusetts, United States of America; 2 Biconcavity Inc., Lilburn, Georgia, United States of America; 3 Yale School of Public Health and Yale School of Medicine, New Haven, Connecticut, United States of America; 4 Frank H. Netter MD School of Medicine, Quinnipiac University, North Haven, Connecticut, United States of America; 5 American Red Cross, Rockville, Maryland, United States of America; George Washington University School of Medicine and Health Sciences, UNITED STATES

## Abstract

**Background:**

Babesiosis is an emerging infectious disease caused by intraerythrocytic *Babesia* parasites that can cause severe disease and death. While blood type is known to affect the mortality of *Plasmodium falciparum* malaria patients, associations between red blood cell (RBC) antigens and *Babesia microti* infection and disease severity are lacking.

**Methods:**

We evaluated RhD and ABO blood types of *Babesia-*infected (18S rRNA reactive) blood donors in 10 endemic states in the Northeastern and northern Midwestern United States. We also assessed possible associations between RhD and ABO blood types and disease severity among hospitalized babesiosis patients in Connecticut.

**Results:**

A total of 768 *Babesia*-infected blood donors were analyzed, of which 750 (97.7%) had detectable *B*. *microti*-specific antibodies. *B*. *microti*-infected blood donors were more likely to be RhD- (OR of 1.22, p-value 0.024) than RhD+ donors. Hospitalized RhD- babesiosis patients were more likely than RhD+ patients to have high peak parasitemia (p-value 0.017), which is a marker for disease severity. No differences in RhD+ blood type were noted between residents of the Northeast (OR of 0.82, p-value 0.033) and the Midwest (OR of 0.74, p-value 0.23). Overall, ABO blood type was not associated with blood donor *B*. *microti* infection, however, *B*. *microti*-infected donors in Maine and New Jersey were more likely to be blood type B compared to non-type B (OR 2.49 [p = 0.008] and 2.07 [p = 0.009], respectively), while infected donors from Pennsylvania were less likely to be type B compared to non-type B (OR 0.32 [p = 0.02]).

**Conclusions:**

People expressing RhD antigen may have a decreased risk of *B*. *microti* infection and babesiosis severity. The association of B antigen with *B*. *microti* infection is less clear because the antigen appeared to be less prevalent in infected Pennsylvania blood donors but more prevalent in Maine and New Jersey infected donors. Future studies should quantify associations between *B*. *microti* genotypes, RBC antigens, and the frequency and severity of *B*. *microti* infection to increase our understanding of human *Babesia* pathogenesis and improve antibody, vaccine, and RBC exchange transfusion strategies.

## Author summary

We found an association between *Babesia microti* infection and both red blood cell RhD and B antigens. Numerous studies have quantified the impact of ABO blood type on *Plasmodium falciparum* (*Pf*) infection, an intraerythrocytic pathogen in the same Apicomplexa phylum as *Babesia*. For example, type O blood has been noted to protect against severe malaria, which may explain why type O is prevalent in *Pf*-endemic regions. Studies of ABO and RhD blood types and *B*. *microti* infection are lacking. The American Red Cross has a large blood donor dataset from *B*. *microti*-endemic states that can be used to study how ABO and RhD blood types are linked to *B*. *microti* infection, as measured by the presence of *B*. *microti* 18S rRNA. Data from 10 *B*. *microti*-endemic states showed that *B*. *microti*-infected blood donors were significantly less likely to be RhD+ (OR 0.82, p-value 0.024) than RhD- donors. A review of babesiosis patients admitted to Yale New Haven Hospital between 2011 and 2021 revealed that RhD+ patients were significantly less likely than RhD- patients to have a high (>5%) peak parasitemia (p = 0.017), which correlates with disease severity. Thus, the RhD antigen on the RBC membrane may decrease the risk of *B*. *microti* infection and disease severity. The blood type B antigen may affect the frequency of *B*. *microti* infection. The odds ratios (ORs) of *B*. *microti* infection in blood donors for blood type B compared to non-type B were 2.49 (p = 0.008) in Maine and 2.07 (p = 0.009) in New Jersey, but 0.32 (p = 0.02) in Pennsylvania. Future studies should quantify the impact of RhD, ABO, and other RBC antigens on *Babesia* infection, and the mechanisms by which they impact RBC invasion and babesiosis severity.

## Introduction

The United States (US) has the highest number of human babesiosis cases worldwide, and the marked increase in cases over the past two decades has led to its classification as an “emerging infectious disease” [1,2]. Babesiosis is caused by Apicomplexan parasites known as *Babesia*, which are unicellular intraerythrocytic organisms. Almost all cases in the US are caused by *Babesia microti* and primarily transmitted to humans by *Ixodes scapularis* ticks. However, *Babesia* can also be acquired via blood transfusion, perinatal transmission, and organ transplantation [3]. *B*. *microti* usually causes a mild to moderate infection but can also present as an asymptomatic infection or severe, life-threatening disease requiring hospital admission. Severe disease often occurs in people aged 50 or older and those who are immunocompromised, including patients with asplenia, malignancy, HIV/AIDS, or on immunosuppressive medication. The mortality rate in immunocompromised patients can exceed 20% [4].

Red blood cells (RBCs or erythrocytes) are essential for the growth and replication of *Babesia* and *Plasmodium* protozoan parasites [5]. These parasites bind to RBC surface antigens, invade, and express adherence antigens on the RBC surface [6]. This can cause severely impaired microvascular blood flow, leading to disease and sometimes death. RBC surface antigens may impact the risk of infection and disease severity. Duffy antigen negative reticulocytes resist *Plasmodium vivax* invasion [7]. Certain *P*. *falciparum (Pf)* strains require sialic acid for invasion [8,9], while basigin (CD147) may be essential for invasion of all *Pf* strains [10]. Both *Pf* and *B*. *divergens* use glycophorins A and B to invade RBCs [11], and *Pf* can also use glycophorin C (Gerbich blood group system) [12]. Blood group O patients have substantial protection against severe *Pf* malaria infection and death due to decreased RBC rosetting (binding of uninfected RBCs to a central parasitized RBC) and vascular endothelial cell adherence (cytoadherence) [13]. This likely explains the high prevalence of blood group O in *Pf* malaria “hot zones.” [14] Little is known about how RBC antigens impact *B*. *microti* invasion and babesiosis complications.

In this study, we sought to determine if RhD and ABO RBC antigens are associated with *B*. *microti* infection, as determined by *B*. *microti* 18S rRNA, or babesiosis disease severity. We calculated the odds ratios (ORs) linking blood type and *B*. *microti* 18S rRNA test reactivity for blood donors in 10 Midwestern and Northeastern *B*. *microti*-endemic states where blood donations are routinely screened for the parasite. We also determined whether RhD and ABO blood types are associated with peak parasitemia and clinical outcomes among babesiosis patients hospitalized for severe disease.

## Methods

### *Babesia*-infected blood donors

American Red Cross (ARC) blood donations were collected between May 2020 and December 2021 in 10 states (Maine, New Hampshire, Vermont, Massachusetts, Connecticut, New York, New Jersey, Pennsylvania, Wisconsin, and Minnesota). ABO and RhD blood type was determined by routine testing performed by the ARC using the PK7400 Automated Microplate System from Beckman Coulter. Testing donated blood for the presence of *Babesia* parasite and *Babesia* antibodies has been previously described [15]. In brief, testing was performed at Creative Testing Solutions (CTS) as a routine operational blood screening. *Babesia* nucleic acid testing (NAT) was performed using the Procleix *Babesia* assay (Grifols Diagnostic Solutions, San Diego, CA) from whole blood lysates. This FDA-licensed assay detects 18S rRNA from *B*. *microti*, *B*. *divergens*, *B*. *duncani*, and *B*. *venatorum* using transcription-mediated amplification (TMA). Samples were tested in pools of 16. If the pool result was reactive, each of the lysates were tested individually to identify the reactive donation. The assay was repeated on a newly prepared lysate to confirm the initial result. Donations that tested reactive in both individual tests were considered repeat reactive (RR); donations that were not repeatedly reactive on the secondary lysate were considered initial reactive (IR). Both IR and RR were considered reactive, and the donors were deferred for two years. *B*. *microti*-specific immunofluorescence using a research assay was performed on all reactive donations.

### Hospitalized babesiosis patients

We analyzed a database of babesiosis patients 18 years and older who were admitted to Yale New Haven Hospital in New Haven, CT, between 2011 and 2021, as previously described [16]. The diagnosis of babesiosis was confirmed by microscopic detection of *Babesia* on blood smears and/or amplification of *B*. *microti* DNA by polymerase chain reaction (PCR) testing.

### Statistical analyses

For blood-donor analyses, contingency tables with chi-square testing were made using GraphPad Prism software, version 9.3.1. For hospitalized patients, contingency tables were made and analyzed in SAS Studio 3.8 using Chi-squared; Fisher’s exact testing was used when the sample count in a given category was <5. P-values less than 0.05 were considered statistically significant.

## Results

### *Babesia*-reactive blood donors

A total of 861,712 *Babesia*-tested blood donations from unique donors were included in this study (Table 1). Of these, 768 individuals (0.089%) were *Babesia* NAT reactive, with 750 (97.7%) positive by a *B*. *microti*-specific antibody assay. ARC data from 10 states were analyzed: Maine (ME), New Hampshire (NH), Vermont (VT), Massachusetts (MA), Connecticut (CT), New York (NY), New Jersey (NJ), and Pennsylvania (PA) in the Northeast region, and Wisconsin (WI) and Minnesota (MN) in the Midwest region. *B*. *microti* is the only *Babesia* species that has been identified in these states, although different genotypic strains exist [5,17,18].

**Table 1:** Comparing the ABO and RhD blood types of *Babesia*-NAT reactive and non-reactive blood donors. CI = confidence interval.

Blood type	A	B	AB	O	RhD+
**Northeast**					
Non-reactive	212,884	68,111	24,670	307,823	496,269
Reactive	235	92	23	336	533
Chi-square	0.06	3.70	0.79	0.39	4.53
p-value	0.81	0.054	0.37	0.531	**0.033**
OR	0.98	1.24	0.83	0.95	0.82
95% CI	0.84–1.15	1.00–1.55	0.55–1.26	0.82–1.11	0.69–0.99
**Midwest**					
Non-reactive	93,128	26,570	10,866	116,892	197,393
Reactive	30	9	3	40	61
Chi-square	0.04	0.005	0.10	0.08	1.47
p-value	0.84	0.94	0.75	0.78	0.23
OR	0.96	1.03	0.83	1.06	0.74
95% CI	0.61–1.50	0.51–2.05	0.26–2.62	0.69–1.64	0.45–1.21
**All 10 States**					
Non-reactive	306,012	94,681	35,536	424,715	693,662
Reactive	265	101	26	376	594
Chi-square	0.36	3.64	1.07	0.04	5.10
p-value	0.55	0.057	0.30	0.84	**0.024**
OR	0.96	1.23	0.81	0.99	0.82
95% CI	0.82–1.11	0.99–1.51	0.55–1.20	0.86–1.14	0.70–0.97

**Fig 1:**
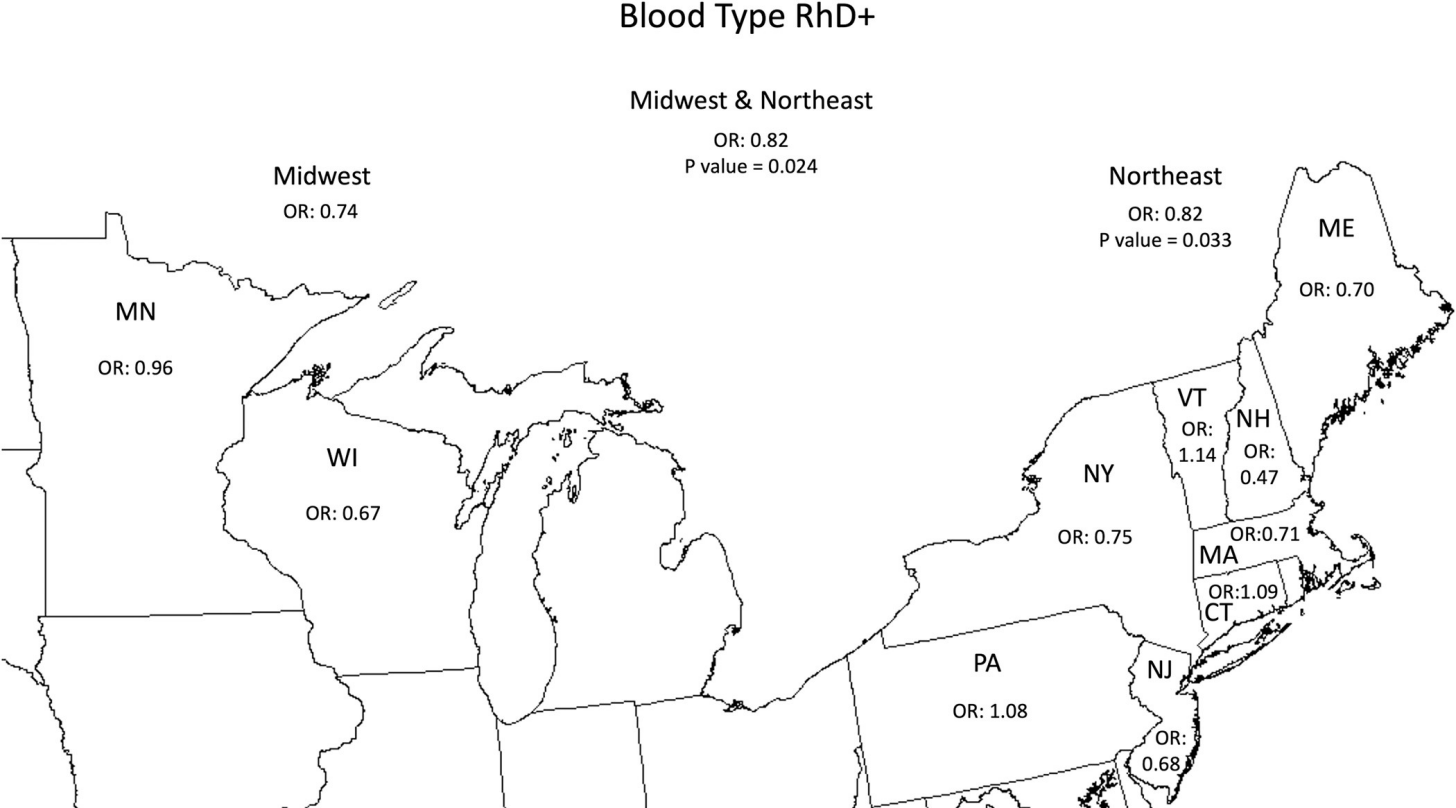
The association of *Babesia*-reactive blood donors with blood type RhD+ compared to RhD-. The associations were statistically significant (p<0.05) in the Northeast and the combined Northeast and Midwest using a chi-square test. This map was cropped from a public domain version created by Brian Szymanski found at Wikimedia Commons website https://commons.wikimedia.org/wiki/File:Usa-state-boundaries-lower48%2B2.png.

Data from all 10 states showed that *Babesia* NAT reactive blood donors were less likely to be RhD+ (OR 0.82, p-value 0.024) than RhD- donors. The Northeast region had an OR of 0.82 (p-value 0.033), while the Midwest had an OR of 0.74 (p-value 0.23). In both regions, the OR for *Babesia* NAT reactivity was decreased in RhD+ donors. No statistically significant association for RhD+ blood type was found for any individual state (Fig 1).

**Fig 2:**
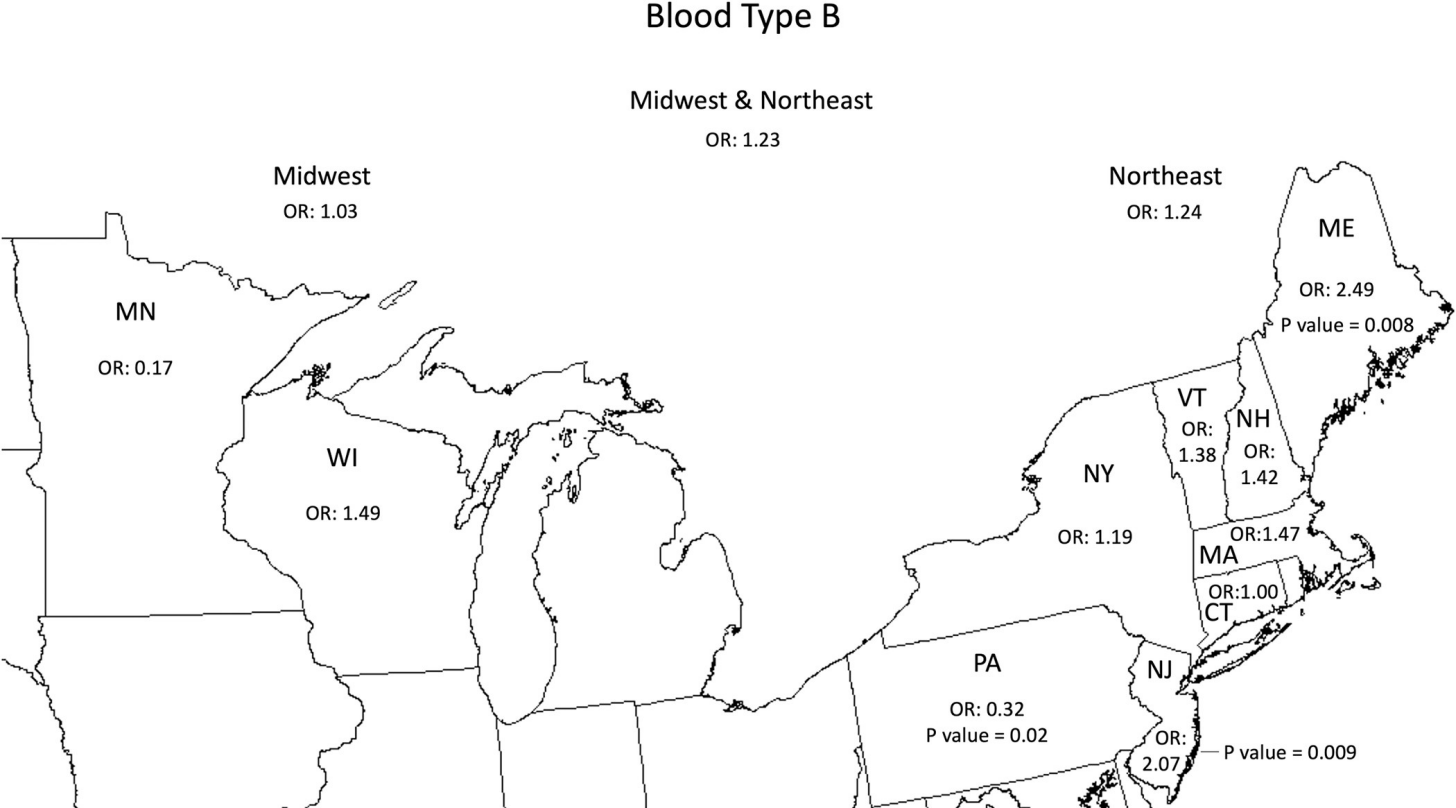
The association of *Babesia*-reactive blood donors with blood type B compared to non-type B. The associations were statistically significant (p<0.05) for PA, NJ, and ME using a chi-square test. This map was cropped from a public domain version created by Brian Szymanski found at Wikimedia Commons website https://commons.wikimedia.org/wiki/File:Usa-state-boundaries-lower48%2B2.png.

The analysis of state-specific data revealed limited associations between ABO antigens and *Babesia* infection. The OR for *Babesia* infection was significantly lower for B blood type donors than for non-B blood donors in Pennsylvania (0.32 [p = 0.02]), whereas the ORs for *Babesia* infection were higher for B blood type donors in Maine and New Jersey (2.49 [p = 0.008] and 2.07 [p = 0.009]), respectively (Fig 2). No other statistically significant results were found at the individual state level (S1–S3 Figs).

### Hospitalized inpatients with babesiosis

Blood types and *Babesiosis* disease severity were assessed in babesiosis patients admitted to Yale New Haven Hospital between 2011 and 2021. Possible associations between RhD or ABO blood types and peak parasitemia, the length of hospital stay, and intensive care unit (ICU) admission were evaluated. Peak parasitemia was defined as the highest percentage of RBCs containing *Babesia* parasites identified on peripheral blood smears during hospitalization. A total of 120 patients were evaluated with the following frequency of blood types: A (n = 47), B (n = 11), AB (n = 6) and O (n = 56). The majority of patients were RhD+ (n = 108, 90.0%) and the rest were RhD- (12, 10.0%).

**Fig 3:**
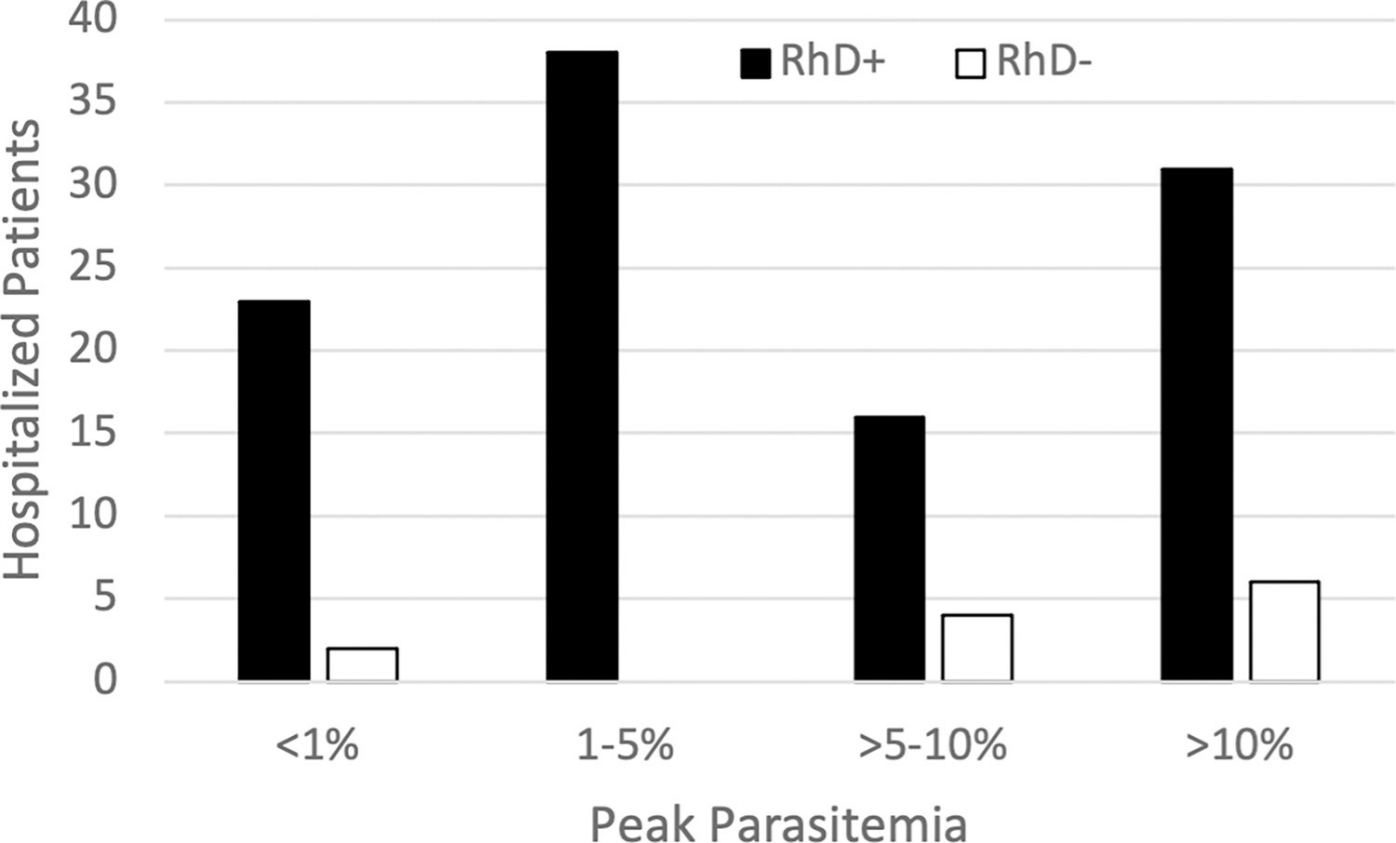
Peak parasitemia percentages in babesiosis patients at Yale New Haven Hospital by RhD blood type. The p-value for Fisher’s exact test was 0.017, indicating a significant association between peak parasitemia and RhD- blood type.

RhD+ patients were less likely (p-value 0.017) to have high parasitemia levels of >5% (Fig 3). There were no significant associations between RhD or ABO blood types and length of hospital stay or intensive care unit admission. A review of 139 hospitalized babesiosis patients in the state of New York found six clinical and laboratory factors that were significantly associated with disease severity. The one with the strongest association was parasitemia levels of 4% or more with a relative risk of 2.48 (p<0.001) [19].

## Discussion

We found that blood donors who have the RhD RBC antigen (RhD+) are less likely to be *Babesia-*infected than donors without the antigen (RhD-). The odds ratio for RhD+ blood donors in the Midwest and Northeast were both less than 1.0, despite differences in *B*. *microti* genotypes in these two regions [20]. Among hospitalized babesiosis patients from Connecticut, those with the RhD+ RBC antigen had lower peak parasitemia (p = 0.017), compared to those who were RhD-. Peak parasitemia has been shown to be strongly associated with disease severity [16]. Thus, data from both our blood donor and hospitalized babesiosis patient populations suggest that the RhD antigen may play a role in inhibiting *B*. *microti* entrance and/or proliferation within RBCs. We found that ABO blood type generally does not correlate with *Babesia* infection or disease, although type B blood donors from Maine and New Jersey were more likely to be *Babesia-*infected while those from Pennsylvania were less likely to be *Babesia-*infected.

The mechanism(s) of RBC antigen enhancement or inhibition of *Babesia* infection and/or disease is unknown. Studies comparing RhD+ and RhD- individuals with *Plasmodium* infection in Africa have yielded inconsistent findings with some studies showing RhD+ individuals more likely to be *Plasmodium* infected and other studies showing RhD+ individuals less likely to be *Plasmodium* infected [21–25]. Differences in results may be due to differences in *Plasmodium* species or genotypes–which were not always accounted for. In one study, *Pf*-parasites and RBCs of four different blood groups were co-cultured. Parasite invasion was less for type B RBCs than for type A and type O RBCs. This appeared to be due, at least in part, to the amount of surface H antigen in each blood group [26]. Different *Babesia* strains have different surface antigens that may bind more or less strongly to B antigens on RBCs. It has been shown that inland and coastal *Babesia* genotypes in Massachusetts differ [20]. Differences in *Babesia* surface antigens may explain why type B blood donors in Maine and New Jersey (coastal states) were more likely to be *Babesia-*infected and those from Pennsylvania (an inland state) were less likely to be infected. Thus, the RBC B antigen may promote invasion for some *B*. *microti* genotypes while resisting invasion for other genotypes.

Establishing a connection between blood type and an infectious disease can be problematic, as illustrated by the often-discrepant findings of research on ABO blood group and SARS-CoV-2 infection and COVID-19 severity. Differing findings may be due, at least in part, to viral genetic variants, which were not always identified in these studies [27–31]. There are several suggested mechanisms of protection in those studies that identified patients with blood type O as having fewer SARS-CoV-2 infections and milder COVID-19 severity. Anti-A and/or anti-B antibodies that are found in patients with type O blood, are thought to neutralize A and/or B antigens on the SARS-CoV-2 viral envelope or S protein [27,32]. Other possible mechanisms of protection involve RBC blood group only indirectly. ABO(H) glycans on the viral S protein or lung epithelial cells may impact viral infection [32], while ABO(H) antigens on von Willebrand factor (vWF) and coagulation factor VIII (FVIII) are known to impact thromboembolic risk, which may impact COVID-19 disease severity [32,33].

Our study has several limitations. The ARC and Yale New Haven Hospital data do not include information about the *Babesia* species or genotypes. The *Babesia* assay used to test blood donors does not distinguish between *B*. *microti*, *B*. *divergens*, *B*. *duncani*, and *B*. *venatorum*, however, *B*. *microti* is the only *Babesia* species found at our study sites. In addition, 97.7% of *Babesia-*reactive blood donors had *B*. *microti*-specific antibodies. The only *Babesia* species that is endemic in Connecticut is *B*. *microti*. The blood-donor analysis did not include percent parasitemia, and blood donors are not exactly representative of the general population. For example, blood donors in the US tend to be healthier than the general population [34], and minorities are underrepresented in the blood-donor pool [35]. Statistically significant associations with the B antigen and *B*. *microti* infection were only found in three states and Pennsylvania had an opposite OR compared to New Jersey and Maine. It is possible that the B antigen does not impact RBC invasion and that *B*. *microti* genotypes do not differ in inland Pennsylvania and coastal New Jersey and Maine. Only 12 of the hospitalized babesiosis patients were RhD- blood type. Finally, more comprehensive RBC genotype and phenotype data could have great value because antigens other than ABO(H) and RhD may be important for parasite invasion and disease severity.

Identifying the mechanisms of RBC blood type risk factors for the development of *Babesia* infection and severe babesiosis may help improve the treatment and prevention of babesiosis. Current antibiotic therapy is effective in most cases but inducible antibiotic resistance can develop in *B*. *microti* during prolonged duration with standard antibiotic regimens in immunocompromised hosts [36–39]. A better understanding of the mechanism of *Babesia* entrance into RBCs might help in the development of antibiotics that impair parasite invasion. RBC exchange transfusion is an adjunct therapy used in severe cases of babesiosis [36,40]. Use of *Babesia-*resistant RBCs might improve the efficacy of exchange transfusion. For example, M- and/or S-antigen negative RBCs show resistance to *B*. *divergens* invasion *in vitro* and have been recently proposed for use in RBC exchange transfusion for the treatment of this type of babesiosis [41]. *B*. *divergens* mortality can exceed 40% in immunocompromised patients and rebound parasitemia can occur after RBC exchange transfusion. Similarly, because type O RBCs offer protection against severe *Pf* malaria, due to decreased rosetting and cytoadherence, they have been proposed for use in RBC exchange transfusion [42].

Additional studies that clarify possible associations between blood types and *Babesia* acquisition and babesiosis mortality are needed. For example, narrowly focused genotyping of the *Babesia* strains isolated from the *Babesia*-reactive type-B blood donors in Pennsylvania, New Jersey, and Maine might be an efficient way to identify different *Babesia* antigens and genotypes, revealing pathogen diversity that might promote development of effective monoclonal antibody combinations and/or a human *Babesia* vaccine.

## Supporting information

S1 Fig The association of *Babesia*-reactive blood donors with blood type A compared to non-type A. The association was not statistically significant using a chi-square test. This map was cropped from a public domain version created by Brian Szymanski found at Wikimedia Commons website https://commons.wikimedia.org/wiki/File:Usa-state-boundaries-lower48%2B2.png.
(TIFF)

S2 Fig The association of *Babesia*-reactive blood donors with blood type AB compared to non-type AB. The association was not statistically significant using a chi-square test. This map was cropped from a public domain version created by Brian Szymanski found at Wikimedia Commons website https://commons.wikimedia.org/wiki/File:Usa-state-boundaries-lower48%2B2.png.
(TIFF)

S3 Fig The association of *Babesia*-reactive blood donors with blood type O compared to non-type O. The association was not statistically significant using a chi-square test. This map was cropped from a public domain version created by Brian Szymanski found at Wikimedia Commons website https://commons.wikimedia.org/wiki/File:Usa-state-boundaries-lower48%2B2.png.
(TIFF)

## References

[1] Kumar A, O’Bryan J, Krause PJ. The Global Emergence of Human Babesiosis. Pathogens. 2021;10(11). Epub 20211106. doi: 10.3390/pathogens10111447 ; PubMed Central PMCID: PMC8623124. 34832603 PMC8623124

[2] Yang Y, Christie J, Köster L, Du A, Yao C. Emerging Human Babesiosis with "Ground Zero" in North America. Microorganisms. 2021;9(2). Epub 20210220. doi: 10.3390/microorganisms9020440 ; PubMed Central PMCID: PMC7923768. 33672522 PMC7923768

[3] Prevention CfDCa. Parasites—Babesiosis 2018. Available from: https://www.cdc.gov/parasites/babesiosis/index.html.

[4] Vannier E, Krause PJ. Human babesiosis. N Engl J Med. 2012;366(25):2397–407. doi: 10.1056/NEJMra1202018 . 22716978

[5] Puri A, Bajpai S, Meredith S, Aravind L, Krause PJ, Kumar S. Babesia microti: Pathogen Genomics, Genetic Variability, Immunodominant Antigens, and Pathogenesis. Front Microbiol. 2021;12:697669. Epub 20210903. doi: 10.3389/fmicb.2021.697669 ; PubMed Central PMCID: PMC8446681. 34539601 PMC8446681

[6] Juillerat A, Lewit-Bentley A, Guillotte M, Gangnard S, Hessel A, Baron B, et al. Structure of a Plasmodium falciparum PfEMP1 rosetting domain reveals a role for the N-terminal segment in heparin-mediated rosette inhibition. Proc Natl Acad Sci U S A. 2011;108(13):5243–8. Epub 20110314. doi: 10.1073/pnas.1018692108 ; PubMed Central PMCID: PMC3069207. 21402930 PMC3069207

[7] Gunalan K, Niangaly A, Thera MA, Doumbo OK, Miller LH. Plasmodium vivax Infections of Duffy-Negative Erythrocytes: Historically Undetected or a Recent Adaptation? Trends Parasitol. 2018;34(5):420–9. Epub 20180309. doi: 10.1016/j.pt.2018.02.006 ; PubMed Central PMCID: PMC6347384. 29530446 PMC6347384

[8] Duraisingh MT, Maier AG, Triglia T, Cowman AF. Erythrocyte-binding antigen 175 mediates invasion in Plasmodium falciparum utilizing sialic acid-dependent and -independent pathways. Proc Natl Acad Sci U S A. 2003;100(8):4796–801. Epub 20030402. doi: 10.1073/pnas.0730883100 ; PubMed Central PMCID: PMC153635. 12672957 PMC153635

[9] Nyarko PB, Tarr SJ, Aniweh Y, Stewart LB, Conway DJ, Awandare GA. Investigating a Plasmodium falciparum erythrocyte invasion phenotype switch at the whole transcriptome level. Sci Rep. 2020;10(1):245. Epub 20200114. doi: 10.1038/s41598-019-56386-y ; PubMed Central PMCID: PMC6959351. 31937828 PMC6959351

[10] Crosnier C, Bustamante LY, Bartholdson SJ, Bei AK, Theron M, Uchikawa M, et al. Basigin is a receptor essential for erythrocyte invasion by Plasmodium falciparum. Nature. 2011;480(7378):534–7. Epub 20111109. doi: 10.1038/nature10606 ; PubMed Central PMCID: PMC3245779. 22080952 PMC3245779

[11] Lobo CA. Babesia divergens and Plasmodium falciparum use common receptors, glycophorins A and B, to invade the human red blood cell. Infect Immun. 2005;73(1):649–51. doi: 10.1128/IAI.73.1.649-651.2005 ; PubMed Central PMCID: PMC538995. 15618210 PMC538995

[12] Maier AG, Duraisingh MT, Reeder JC, Patel SS, Kazura JW, Zimmerman PA, et al. Plasmodium falciparum erythrocyte invasion through glycophorin C and selection for Gerbich negativity in human populations. Nat Med. 2003;9(1):87–92. Epub 20021209. doi: 10.1038/nm807 ; PubMed Central PMCID: PMC3728825. 12469115 PMC3728825

[13] Rowe JA, Handel IG, Thera MA, Deans AM, Lyke KE, Koné A, et al. Blood group O protects against severe Plasmodium falciparum malaria through the mechanism of reduced rosetting. Proc Natl Acad Sci U S A. 2007;104(44):17471–6. Epub 20071024. doi: 10.1073/pnas.0705390104 ; PubMed Central PMCID: PMC2077280. 17959777 PMC2077280

[14] Cserti CM, Dzik WH. The ABO blood group system and Plasmodium falciparum malaria. Blood. 2007;110(7):2250–8. Epub 20070514. doi: 10.1182/blood-2007-03-077602 . 17502454

[15] Tonnetti L, Dodd RY, Foster G, Stramer SL. Babesia blood testing: the first-year experience. Transfusion. 2022;62(1):135–42. Epub 20211102. doi: 10.1111/trf.16718 . 34726279

[16] O’Bryan J, Gokhale A, Hendrickson JE, Krause PJ. Parasite burden and red blood cell exchange transfusion for babesiosis. J Clin Apher. 2021;36(1):127–34. Epub 20201112. doi: 10.1002/jca.21853 . 33179803 PMC9517950

[17] Baniecki ML, Moon J, Sani K, Lemieux JE, Schaffner SF, Sabeti PC. Development of a SNP barcode to genotype Babesia microti infections. PLoS Negl Trop Dis. 2019;13(3):e0007194. Epub 20190325. doi: 10.1371/journal.pntd.0007194 ; PubMed Central PMCID: PMC6448979. 30908478 PMC6448979

[18] Carpi G, Walter KS, Mamoun CB, Krause PJ, Kitchen A, Lepore TJ, et al. Babesia microti from humans and ticks hold a genomic signature of strong population structure in the United States. BMC Genomics. 2016;17(1):888. Epub 20161107. doi: 10.1186/s12864-016-3225-x ; PubMed Central PMCID: PMC5100190. 27821055 PMC5100190

[19] White DJ, Talarico J, Chang HG, Birkhead GS, Heimberger T, Morse DL. Human babesiosis in New York State: Review of 139 hospitalized cases and analysis of prognostic factors. Arch Intern Med. 1998;158(19):2149–54. doi: 10.1001/archinte.158.19.2149 . 9801183

[20] Goethert HK, Molloy P, Berardi V, Weeks K, Telford SR 3rd. Zoonotic Babesia microti in the northeastern U.S.: Evidence for the expansion of a specific parasite lineage. PLoS One. 2018;13(3):e0193837. Epub 20180322. doi: 10.1371/journal.pone.0193837 ; PubMed Central PMCID: PMC5864094. 29565993 PMC5864094

[21] Alemu G, Mama M. Assessing ABO/Rh Blood Group Frequency and Association with Asymptomatic Malaria among Blood Donors Attending Arba Minch Blood Bank, South Ethiopia. Malar Res Treat. 2016;2016:8043768. Epub 20160127. doi: 10.1155/2016/8043768 ; PubMed Central PMCID: PMC4748098. 26925291 PMC4748098

[22] Jeremiah ZA, Jeremiah TA, Emelike FO. Frequencies of some human genetic markers and their association with Plasmodium falciparum malaria in the Niger Delta, Nigeria. J Vector Borne Dis. 2010;47(1):11–6. . 20231768

[23] Mukhtar IG, Rahmat S, Salisu AI. RELATIONSHIP BETWEEN ABO AND Rh D BLOOD GROUP PHENOTYPES AND MALARIA AMONG A POPULATION OF UNDERGRADUATE STUDENTS IN KANO, NIGERIA. FUDMA JOURNAL OF SCIENCES. 2020;4(1):133–7.

[24] Onanuga A LA. Association of ABO blood group and Plasmodium falciparum malaria among Children in the Federal Capital Territory, Nigeria. African Journal of Biomedical Research. 2016;19(1).

[25] Tonen-Wolyec S, Batina-Agasa S. High susceptibility to severe malaria among patients with A blood group versus those with O blood group: A cross-sectional study in the Democratic Republic of the Congo. Trop Parasitol. 2021;11(2):97–101. Epub 20211020. doi: 10.4103/tp.TP_87_20 ; PubMed Central PMCID: PMC8579772. 34765530 PMC8579772

[26] Pathak V, Colah R, Ghosh K. Correlation between ’H’ blood group antigen and Plasmodium falciparum invasion. Ann Hematol. 2016;95(7):1067–75. Epub 20160412. doi: 10.1007/s00277-016-2663-5 . 27071756

[27] Gutiérrez-Valencia M, Leache L, Librero J, Jericó C, Enguita Germán M, García-Erce JA. ABO blood group and risk of COVID-19 infection and complications: A systematic review and meta-analysis. Transfusion. 2022;62(2):493–505. Epub 20211119. doi: 10.1111/trf.16748 ; PubMed Central PMCID: PMC8661771. 34773411 PMC8661771

[28] Zhao J, Yang Y, Huang H, Li D, Gu D, Lu X, et al. Relationship Between the ABO Blood Group and the Coronavirus Disease 2019 (COVID-19) Susceptibility. Clinical Infectious Diseases. 2021;73(2):328–31. doi: 10.1093/cid/ciaa1150 32750119 PMC7454371

[29] Dzik S, Eliason K, Morris EB, Kaufman RM, North CM. COVID-19 and ABO blood groups. Transfusion. 2020;60(8):1883–4. Epub 20200801. doi: 10.1111/trf.15946 ; PubMed Central PMCID: PMC7323215. 32562280 PMC7323215

[30] Wu Y, Feng Z, Li P, Yu Q. Relationship between ABO blood group distribution and clinical characteristics in patients with COVID-19. Clinica Chimica Acta. 2020;509:220–3. doi: 10.1016/j.cca.2020.06.026 32562665 PMC7832938

[31] Solmaz İ, Araç S. ABO blood groups in COVID-19 patients; Cross-sectional study. International Journal of Clinical Practice. 2021;75(4):e13927. doi: 10.1111/ijcp.13927 33296536 PMC7883261

[32] Goel R, Bloch EM, Pirenne F, Al-Riyami AZ, Crowe E, Dau L, et al. ABO blood group and COVID-19: a review on behalf of the ISBT COVID-19 Working Group. Vox Sang. 2021;116(8):849–61. Epub 20210212. doi: 10.1111/vox.13076 ; PubMed Central PMCID: PMC8014128. 33578447 PMC8014128

[33] O’Sullivan JM, Ward S, Fogarty H, O’Donnell JS. More on ’Association between ABO blood groups and risk of SARS-CoV-2 pneumonia’. Br J Haematol. 2020;190(1):27–8. Epub 20200601. doi: 10.1111/bjh.16845 ; PubMed Central PMCID: PMC7276715. 32420611 PMC7276715

[34] O’Brien SF, Drews SJ, Lewin A, Russell A, Davison K, Goldman M. How do we decide how representative our donors are for public health surveillance? Transfusion. 2022. Epub 20221004. doi: 10.1111/trf.17140 . 36193865

[35] Shaz BH, James AB, Hillyer KL, Schreiber GB, Hillyer CD. Demographic Patterns of Blood Donors and Donations in a Large Metropolitan Area. Journal of the National Medical Association. 2011;103(4):351–7. doi: 10.1016/s0027-9684(15)30316-3 21805814

[36] Krause PJ, Auwaerter PG, Bannuru RR, Branda JA, Falck-Ytter YT, Lantos P, Lavergne V, Meissner C, Osani MC, Rips JG, Sood SK, Vannier E, Vaysbrot EE, Wormser G. Clinical Practice Guidelines by the Infectious Diseases Society of America (IDSA): 2020 Guideline on Diagnosis and Management of Babesiosis. Clin Infect Dis, 2021 Jan 27;72(2):185–189. doi: 10.1093/cid/ciab050 33501959

[37] Lemieux JE, Tran AD, Freimark L, et al. A global map of genetic diversity in Babesia microti reveals strong population structure and identifies variants associated with clinical relapse. Nat Microbiol 2016; 1:16079. doi: 10.1038/nmicrobiol.2016.79 27572973 PMC5563076

[38] Simon MS, Westblade LF, Dziedziech A, Visone JE, Furman RR, Jenkins SG, et al. Clinical and Molecular Evidence of Atovaquone and Azithromycin Resistance in Relapsed Babesia microti Infection Associated With Rituximab and Chronic Lymphocytic Leukemia. Clinical Infectious Diseases. 2017;65(7):1222–5. doi: 10.1093/cid/cix477 28541469 PMC6248624

[39] Rogers R, Krause PJ, Norris AM, Ting MH, Nagami EH, Cilley B, et al. Broad Antimicrobial Resistance in a Case of Relapsing Babesiosis Successfully Treated With Tafenoquine. Clinical Infectious Diseases. 2022:ciac473. doi: 10.1093/cid/ciac473 35684960

[40] Padmanabhan A, Connelly-Smith L, Aqui N, Balogun RA, Klingel R, Meyer E, et al. Guidelines on the Use of Therapeutic Apheresis in Clinical Practice—Evidence-Based Approach from the Writing Committee of the American Society for Apheresis: The Eighth Special Issue. J Clin Apher. 2019;34(3):171–354. doi: 10.1002/jca.21705 . 31180581

[41] Jajosky RP, Jajosky AN, Jajosky PG. Optimizing exchange transfusion for patients with severe Babesia divergens babesiosis: Therapeutically-Rational Exchange (T-REX) of M antigen-negative and/or S antigen-negative red blood cells should be evaluated now. Transfus Clin Biol. 2019;26(1):76–9. Epub 20181019. doi: 10.1016/j.tracli.2018.10.001 . 30447802 PMC11702889

[42] Jajosky RP, Jajosky RP, Jajosky PG, Jajosky AN, Jajosky PG. Can therapeutically-rational exchange (T-REX) of type-O red blood cells (RBCs) benefit Plasmodium falciparum malaria patients? Transfus Apher Sci. 2019;58(3):344–5. Epub 20190510. doi: 10.1016/j.transci.2019.04.024 . 31109818 PMC11753622

