# Supplementary figures and images for: The impact of ABO and RhD blood types on Babesia microti infection
Source: PLoS Negl Trop Dis. 2023 Jan 25;17(1):e0011060. doi: 10.1371/journal.pntd.0011060 (PMC9901808; doi:10.1371/journal.pntd.0011060)

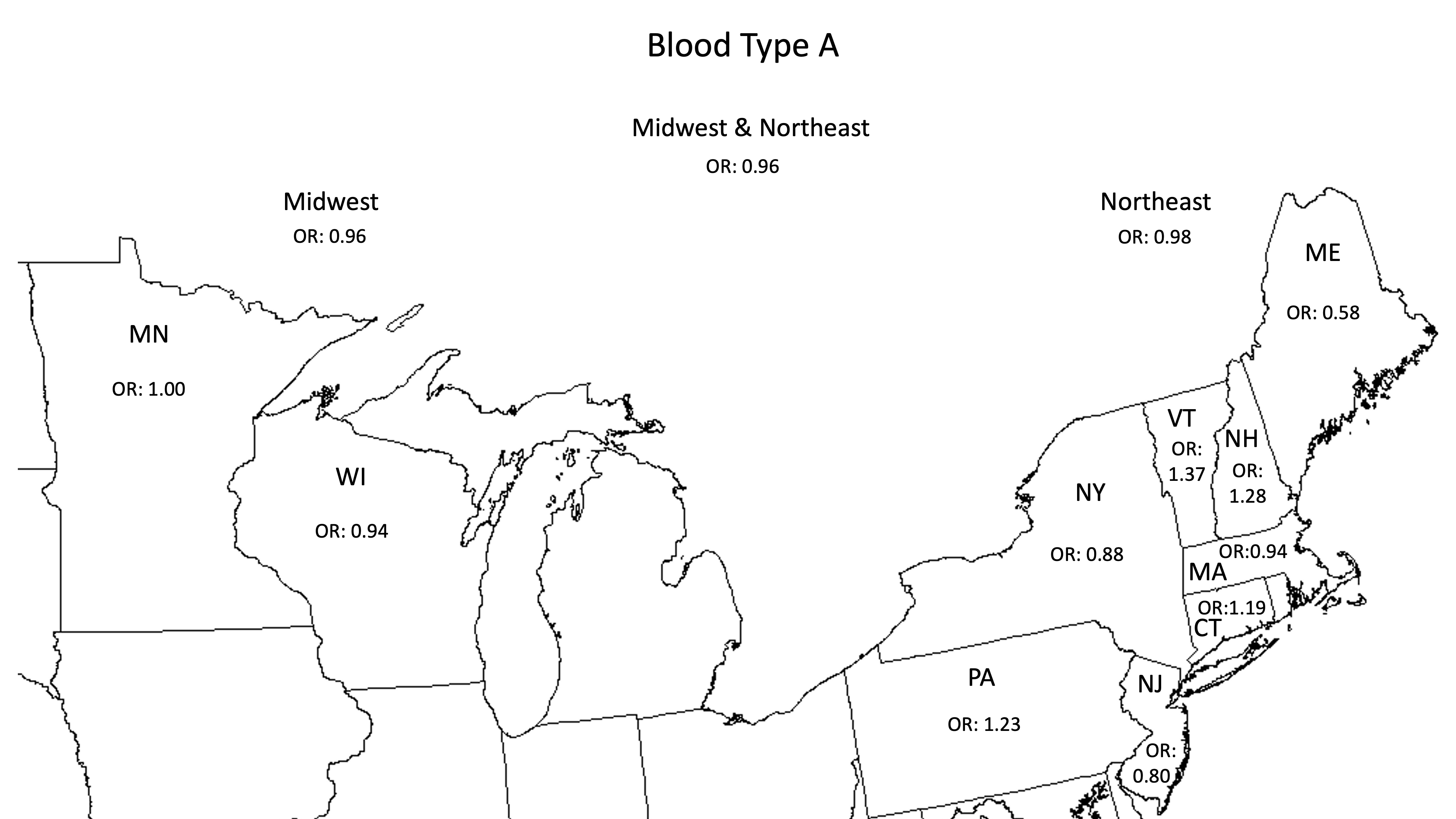

Supplement: S1 Fig — The association was not statistically significant using a chi-square test. This map was cropped from a public domain version created by Brian Szymanski found at Wikimedia Commons website https://commons.wikimedia.org/wiki/File:Usa-state-boundaries-lower48%2B2.png. (TIFF) [file pntd.0011060.s001.tiff]

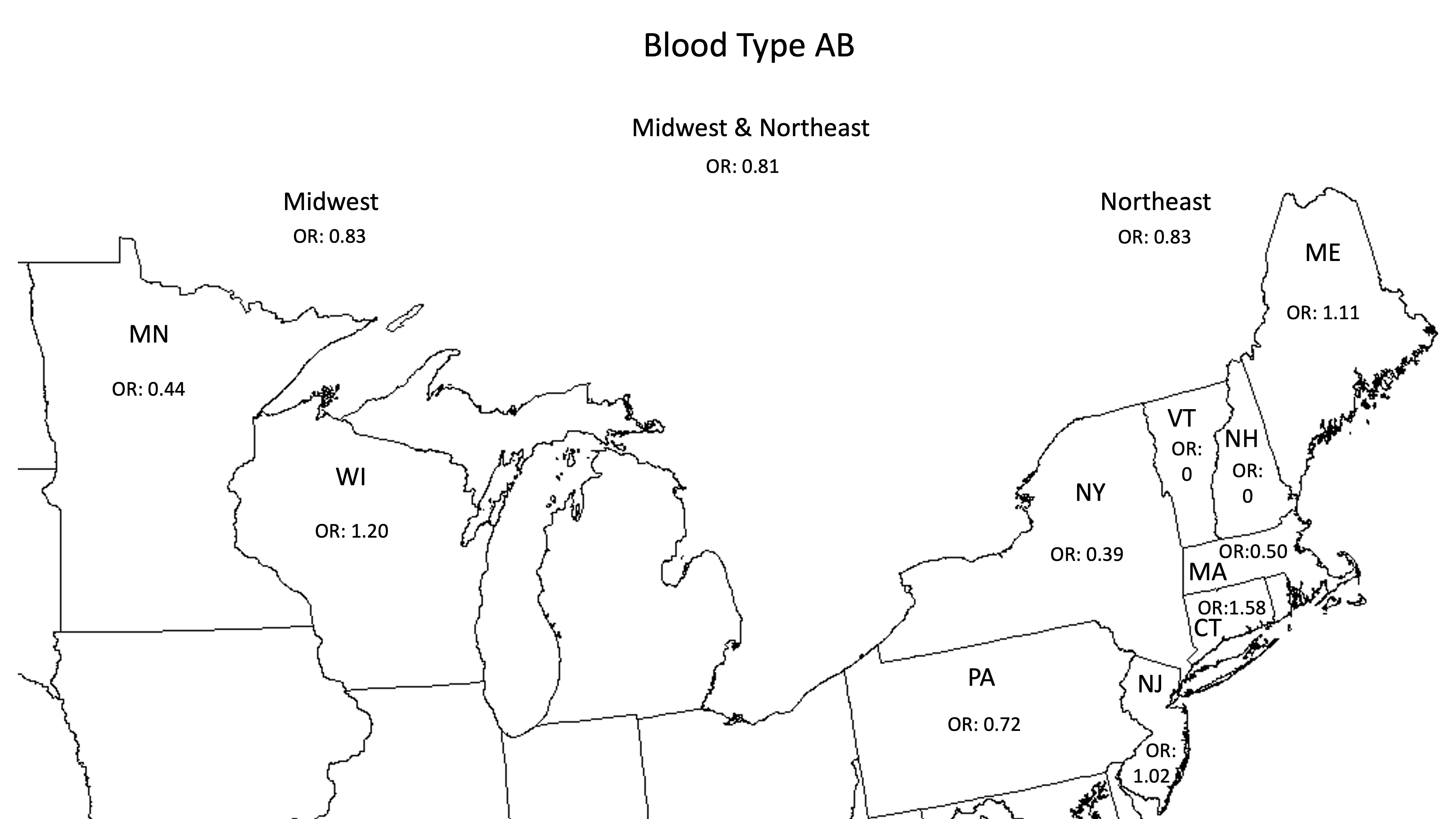

Supplement: S2 Fig — The association was not statistically significant using a chi-square test. This map was cropped from a public domain version created by Brian Szymanski found at Wikimedia Commons website https://commons.wikimedia.org/wiki/File:Usa-state-boundaries-lower48%2B2.png. (TIFF) [file pntd.0011060.s002.tiff]

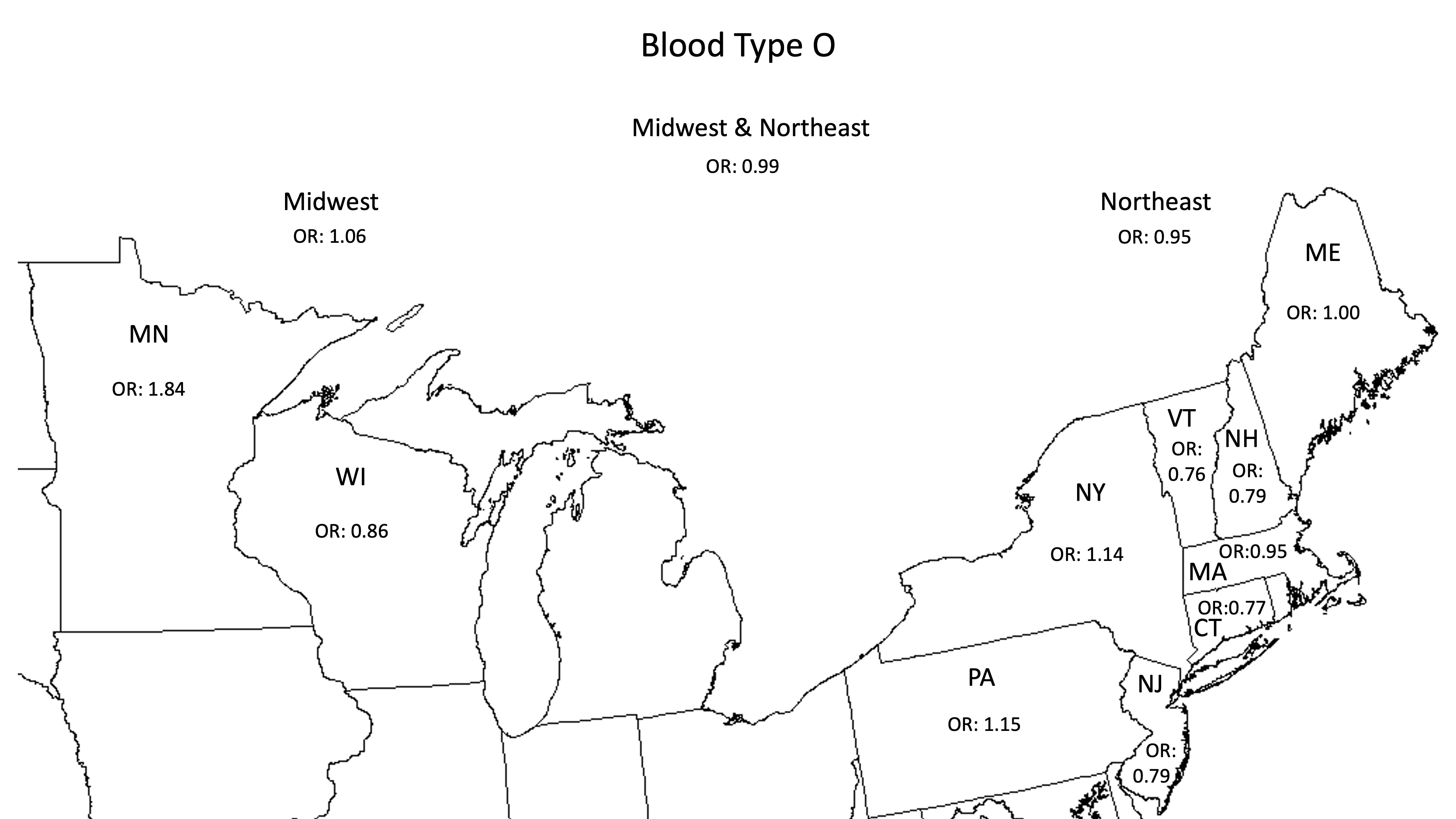

Supplement: S3 Fig — The association was not statistically significant using a chi-square test. This map was cropped from a public domain version created by Brian Szymanski found at Wikimedia Commons website https://commons.wikimedia.org/wiki/File:Usa-state-boundaries-lower48%2B2.png. (TIFF) [file pntd.0011060.s003.tiff]
